# Influence of Selected Factors on Biofilm Formation by *Salmonella enterica* Strains

**DOI:** 10.3390/microorganisms9010043

**Published:** 2020-12-25

**Authors:** Agata Białucha, Eugenia Gospodarek-Komkowska, Joanna Kwiecińska-Piróg, Krzysztof Skowron

**Affiliations:** Department of Microbiology, Nicolaus Copernicus University in Toruń, Ludwik Rydygier Collegium Medicum, 9 M. Skłodowskiej-Curie Street, 85-094 Bydgoszcz, Poland; kolsut@cm.umk.pl (A.B.); gospodareke@cm.umk.pl (E.G.-K.); j.kwiecinska@cm.umk.pl (J.K.-P.)

**Keywords:** *Salmonella* spp., biofilm, glucose, bile, sub-MIC, ampicillin

## Abstract

Biofilm formed by *S. enterica* on the surface of gallstones or biomaterials promotes the development and spread of chronic infection. The aim of the study was to assess biofilm formation on the surface of polystyrene depending on nutritional conditions and the effect of 0.5, 1.0, and 2.0% glucose and 3.0% bile and sub-inhibitory concentrations of ampicillin on biofilm formation of *S. enterica*. Sixty-nine clinical strains of *S. enterica* isolated from feces (92.8%) and blood (7.2%) collected from patients (66.7%) and carriers (33.3%) were used in the study. Assessment of forming 24-h biofilm by these strains was performed on the surface of polystyrene 96-well plates at 37 °C. In this study, it was indicated that 1.0% glucose and 3.0% bovine bile inhibit biofilm formation. Biofilm formation was inhibited in all examined sub-MIC of ampicillin. Biofilm formation is varied in different conditions, depending on the serovar.

## 1. Introduction

*Salmonella enterica* is the prevailing bacterial etiological factor of food poisonings and gastrointestinal tract infections in the world. Main sources of these bacteria include products of animal origin and animal feces which must be subjected to hygienization in order to provide sanitary and hygienic safety [1,2]. Both patients and carriers play an important role in the transmission of these pathogens. Outbreaks of infections can be maintained in the gall bladder, bile ducts, mesentery lymph nodes, liver, or urinary tract. *Salmonella* spp. is maintained in the gallbladder in carriers for the longest time, even for many years [3,4,5,6].

*Salmonella* spp. is bacteria with the ability to adhere and form a biofilm. Biofilm formed on the surface of gallstones or biomaterials favors the development and maintenance of chronic infection. Biofilm formation is caused by such factors as the type of surface to which microorganisms adhere, pH and temperature of the environment, the presence, amount and kind of nutrients, and antimicrobial substances. The biofilm structure impedes the penetration of antimicrobial compounds into its inner layers, limiting their action on the surface layer. Therefore, treatment of infections with the participation of biofilm formed by *Salmonella* spp. has limited effectiveness [7,8]. It is necessary to seek effective methods for its prevention and eradication.

The aim of this study was the formation of 24-h biofilm on the surface of polystyrene in *S. enterica* strains, and the effect of glucose, bile salts, and sub-MIC concentrations of ampicillin on this process.

## 2. Materials and Methods

### 2.1. Strains of S. enterica

The study covered 69 strains of *S. enterica* isolated at the Clinical Microbiology Department of the Antoni Jurasz University Hospital no. 1 in Bydgoszcz, Poland from patients (46, 66.7%) and carriers (23, 33.3%). Strains were obtained from feces (92.8%) and blood (7.2%) samples. All strains isolated from carriers were collected from stool samples. Amongst strains isolated from patients, strains were obtained from feces (41, 89.1%) and from blood samples (5, 10.9%).

The origin and type of material from which the studied strains were examined are presented in Table 1.

Investigation strains were stored at −80 °C (Thermo Scientific, Waltham, MA, USA) in Tryptic Soy Broth (Becton Dickinson, Franklin Lakes, NJ, USA) supplemented with 20% glycerol (Avantor, Gliwice, Poland).

### 2.2. Identification of Strains

Strains of *Salmonella* spp. were identified based on the morphology of colonies on Xylose-Lysine-Desoxycholate Agar (XLD, BioMérieux, Marcy-l’Étoile, France), results of biochemical reactions (Vitek2^®^ Compact), and antigenic structure according to the White-Kauffman-Le Minor scheme (SIT EnTy, Immunolab, Gdańsk, Poland). Amongst all included *S. enterica* strains, 59 (85.5%) were found as *S*. Enteritidis, 5 (7.2%) as *S*. Infantis, 3 (4.4%) as *S*. Typhimurium, and 2 (2.9%) as *S*. Mbandaka (Table 1).

### 2.3. Preparation of Bacterial Suspension

The *S. enterica* strain was cultured on MacConkey Agar (MCA; Becton Dickinson). Bacterial inocula were prepared in Tryptic Soy Broth (TSB; Becton Dickinson), which was incubated at 37 °C. Next, overnight culture was centrifugated, and the precipitate was washed using a sterile Phosphate Buffered Saline (PBS, BTL) with pH 7.2. After re-centrifugation of the bacterial suspension, the precipitate was diluted with sterile TSB, Tryptic Soy Broth without Dextrose (TSB BD; Becton Dickinson) or Mueller-Hinton II Broth (MHB; Becton Dickinson) to obtain a suspension of bacteria (a density 0.5 McFarland standards) which were used in next steps of examination.

### 2.4. Assessment of Biofilm Formation

We performed the 96-well plate assay with crystal violet (CV) described by Christensen et al. [9], with some modifications. The 200 μL of suspensions prepared in TSB as described in Section 2.3 were placed into wells of polystyrene 96-well plates (Profilab, Warsaw, Poland), in three replications of each variant. The negative control was the sterile medium in three replications. Biofilms were cultured at 37 °C. After 24 h, biofilm was washed five times using PBS to remove sessile cells. The mature biofilm formed in polystyrene wells was dried for 20 min at 37 °C. Next, dry biofilm was fixed using 200 µL 96% ethyl alcohol (Avantor). Fixed biofilm was stained using 200 µL 2.0% crystal violet (Avantor). CV, bound with attached biofilm cells and extracellular matrix, was dissolved in 96% ethyl alcohol. The absorbance of dissolved CV was measured using a spectrophotometer (BioTek, Winooski, VT, USA) by λ = 540 nm. Results were analyzed and archived using the KC4 program (BioTek, Winooski, VT, USA). To assess biofilm forming for each strain and negative control, the arithmetic mean of absorbance and standard deviation were used. The threshold value of absorbance (T) was proof of the biofilm forming and was defined as the sum of the arithmetic mean of negative control and a triple value of its standard deviation (T = x_nc_ + 3δ). A value below the calculated sum was recognized as a lack of biofilm. Weak biofilm was determined when the value of the sum was between T and 2T, moderate biofilm—between 2T and 4T, and strong—for a value higher than 4T.

### 2.5. Assessment of the Effect of Glucose and Bile on Biofilm Formation

A 150 μL bacterial suspension and 50 μL of aqueous solution of glucose (BTL) or bovine bile (BTL) were added to each of the three next wells of 96-wells plates. Final glucose concentrations amounted to 0.5, 1.0, and 2.0%, whereas bile concentrations were 3.0%. The negative control was the sterile medium and the medium with glucose or bile in three replications. The positive control was a bacterial culture in the medium non-supplemented by glucose or bile.

Incubation conditions of the bacterial culture and further activities aimed at fixation, staining, and reading the Optical Density (OD) were conducted according to the description above. Assessment of the effect of glucose and bile on biofilm formation was performed by comparing the OD values of samples with the OD values of the positive controls. The studied strains of *S. enterica* were classified as in the subsection assessment of biofilm formation.

### 2.6. Assessment of the MIC Value of Ampicillin

The value of the Minimal Inhibitory Concentration (MIC) of ampicillin for the examined strains was assessed using strips with antibiotic concentration gradient (BioMérieux) according to the manufacturer instructions and results were interpreted according to recommendations of the European Committee on Antimicrobial Susceptibility Testing, (EUCAST, 2019) [10] for Enterobacterales.

### 2.7. Assessment of the Sub-MIC Values of Ampicillin on Biofilm Formation

A total of 100 μL of sterile MHB and 100 μL of the antibiotic solution were introduced to each of the three next wells of 96-well plates. Final ampicillin concentrations were: 0.125 MIC, 0.25 MIC, and 0.5 MIC. The positive control was a bacterial culture in MHB without the antibiotic. The negative control included MHB and MHB with ampicillin. Plates were placed in a humid chamber at 35 °C for 24 h. After this time, the plates were washed three times with sterile deionized water and dried. Next, 100 μL of the sterile TSB medium and 100 μL of the sterile solution of 0.1% 2,3,5-triphenyltetrazolium chloride (TTC; Avantor) was introduced into each plate well and incubated for two hours at 37 °C. Next, the solution was removed from wells, and the attached polystyrene biofilm was washed five times by using PBS. In the next step, formazan was dissolved in 96% ethyl alcohol.

The OD reading was made at the wavelength λ = 470 nm. Formazan is found only in active (living) bacteria cells as an indicator of metabolic activity. For this reason, to compare results of formazan absorbance, which is strongly strain-dependent, we introduced the biofilm inhibition ratio according to the formula:

$$\frac{\mathrm{Absorbance}\;\mathrm{Control}\left(+\right)-\mathrm{Absorbance}\;\mathrm{Sample}}{\mathrm{Absorbance}\;\mathrm{Control}\left(+\right)}$$

### 2.8. Statistical Analysis

Statistical analysis was performed in the program STATISTICA 13.0 PL (StatSoft, Inc., Tulsa, OK, USA). The distribution of the recorded values was not consistent with the normal distribution. For this reason, the significance of differences was assessed using the Wilcoxon test to compare the results of related variables and the post-hoc Bonferroni test at the significance level α = 0.05.

## 3. Results

Based on the obtained results, 42 (60.9%) strains of S. enteritidis, 5 (7.2%) of S. infantis, 3 strains of S. typhimurium, and 2 strains of S. mbandaka were identified.

### 3.1. Biofilm Formation

Amongst 69 examined strains of *S. enterica*, all were able to form biofilm on polystyrene (Table 2). Based on the criteria of biofilm formation intensity described in the previous section, the highest percentage of strains formed weak biofilm (36, 52.2%). Amongst *S.* Enteritidis, *S.* Infantis, and *S.* Typhimurium strains, most strains (31, 52.5%; 3, 60.0%; 3, 66.7%, respectively) formed weak biofilm. Both strains (100.0%) of *S.* Mbandak formed a moderate biofilm (Table 2). No statistically significant differences were observed in results obtained for strains isolated from sick people or from carriers.

### 3.2. Effect of Glucose on Biofilm Formation

**Figure 1:**
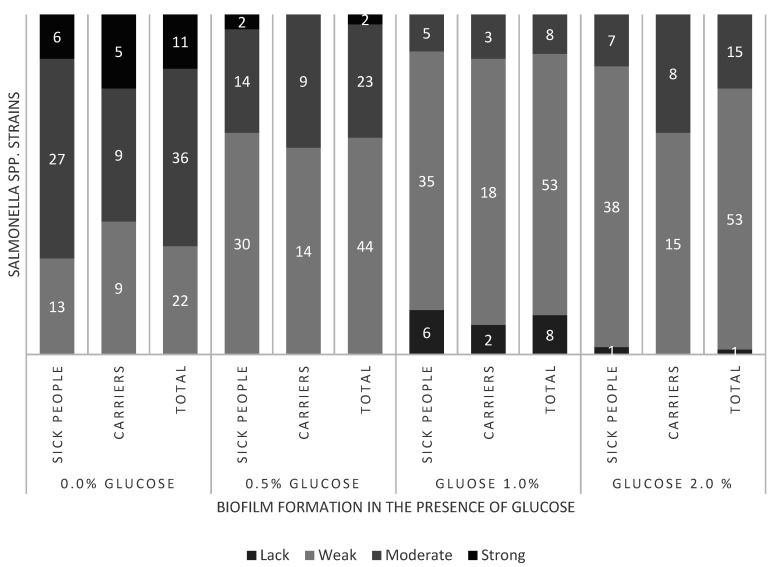
Biofilm of *S. enterica* strains (*n* = 69) in various glucose concentrations.

Based on the mean absorbance values of samples, it was shown that all *S. enterica* strains formed biofilm in TSB BD (control) and TSB BD with an addition of 0.5% glucose (Figure 1). In comparison with the control, in TSB BD with 0.5% glucose, there was a decrease in the number of moderate biofilm-forming (from 36; 52.2% to 23; 33.3%) and strong biofilm-forming strains (from 11; 15.9% to 2.9%), whereas the number of weak biofilm-forming strains increased (from 22; 31.9% to 44; 63.8%). No strong biofilm-forming strains were shown in culture media with the addition of 1.0 and 2.0% glucose. In these growth conditions, weak biofilm-forming strains were found most frequently (53; 76.8%). Moreover, eight (11.6%) strains *S. enterica* stopped forming biofilm in the environment of 1.0% glucose (Figure 1).

The mean absorbance value for the examined strains isolated from patients was the lowest in the TSB medium supplemented with 1.0% glucose (0.0360), and the highest in TSB without glucose (0.1184). An analogous tendency was observed for the strains isolated from carriers, whereas mean absorbance values stayed within the range 0.0362 to 0.1164 (Supplementary Figure S1). It was indicated that in relation to the control (TSB BD), adding glucose significantly (*p* ≤ 0.05) reduced biofilm formation by *S. enterica* strains. Additionally, statistically significant differences (*p* ≤ 0.05) were shown in absorbance values at glucose concentrations equal to 0.5% and 1.0%. It was recorded that after adding glucose, *S. enterica* strains isolated from carriers formed stronger biofilm than the strains isolated from patients, but the indicated differences were not statistically significant (*p* > 0.05).

### 3.3. Effect of Bile on Biofilm Formation

**Figure 2:**
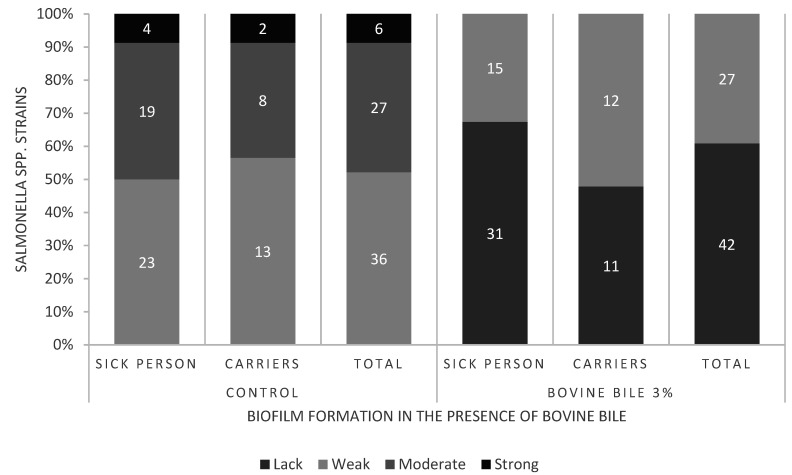
Biofilm of *S. enterica* strains (*n* = 69) in TSB (control) and TSB medium with 3.0% bovine bile.

Based on the mean absorbance values of samples it was shown that among the 69 studied *S. enterica* strains, after adding 3.0% bovine bile to the TSB, no strains forming strong and medium biofilm were found (Figure 2). In these culture conditions 27 (39.1%) strains formed weak biofilm, and the other 42 (60.9%) did not form biofilm. Of *S. enterica* strains isolated from patients, most strains did not form biofilm (31; 67.4%) and the other strains formed weak biofilm (15; 32.6%). Strains isolated from carriers formed weak biofilm (52.2%) and did not form biofilm (47.8%) in TSB containing 3.0% bovine bile.

Regardless of the origin of the studied *S. enterica* strains, the mean value of absorbance for the strains was lower in the TSB medium with 3.0% bovine bile, and higher in TSB (Supplementary Figure S2). The differences were statistically significant (*p* ≤ 0.05).

### 3.4. Assessment of the MIC Value of Ampicillin

**Figure 3:**
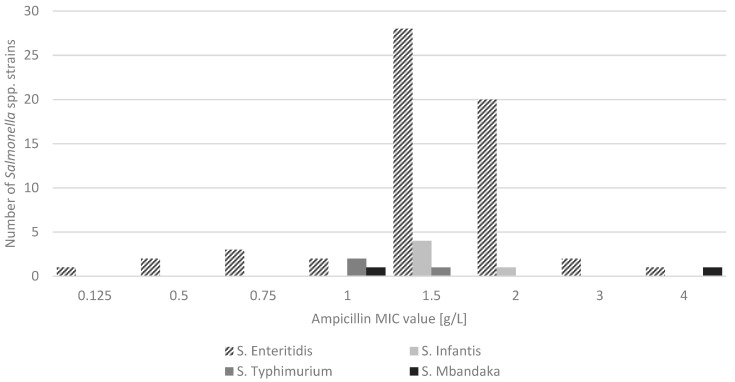
Ampicillin MIC values for *S. enterica* strains (*n* = 69).

Obtained values in Salmonella spp. were in the range 0.125 to 4.0 g/L (Figure 3). On the base of the breakpoints for Enterobacterales (according to EUCAST recommendation), we found that all S. enterica strains were susceptible to ampicillin. Values of MIC below 1 g/L were observed only in six (8.7%) S. Enteritidis strains.

### 3.5. Effect of Sub-MICs of Ampicillin on Biofilm Formation

**Figure 4:**
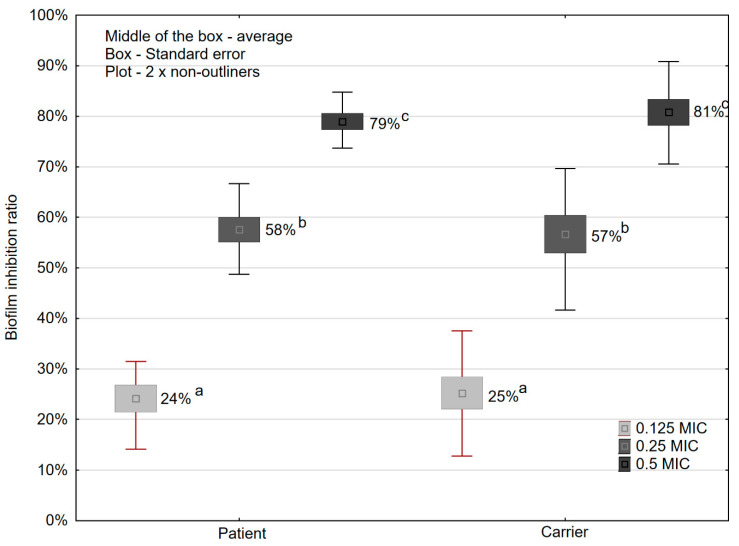
*S. enterica* (*n* = 69) biofilm inhibition ratio in the presence of ampicillin sub-MICs. (a, b, c, values marked with different letters differ statistically significantly (*p* ≤ 0.05)**.**

The ability of sub-MICs ampicillin to inhibit biofilm formation by *S. enterica* strains was described by the biofilm inhibition ratio. Ratio values were the lowest for 0.125 MIC of ampicillin (24.13% for strains isolated from patients and 25.12% for strains isolated from carriers). The highest ratio values were obtained in medium supplemented with 0.5 MIC of ampicillin (78.96% and 80.79% for patients and carriers, respectively). The addition of 0.125 MIC, 0.25 MIC, and 0.5 MIC of ampicillin significantly (*p* ≤ 0.05) inhibited film formation by *S. enterica* strains in relation to the control (MHB without ampicillin (Figure 4). We observed similar ratio values for 0.125 MIC, 0.25 MIC, and 0.5 MIC amongst strains obtained from carriers than amongst those collected from feces of sick people (Figure 4).

## 4. Discussion

*Salmonella enterica* is an intracellular human and animal pathogen. It belongs to the most frequent bacterial etiological agents of gastrointestinal infections in humans [11].

*Salmonella* spp. have the ability to form biofilm both on animate and inanimate surfaces [12]. Biofilm formation is influenced by many factors that can change the expression of genes and their products essential in the process of biofilm formation. In this study, we found that all *S. enterica* strains are able to form biofilm on polystyrene after a 24-h incubation in standard growth conditions. This ability is strongly dependent on biofilm growth conditions. Biofilm formation by the studied strains of *S. enterica* on the surface of polystyrene was limited in the presence of glucose. Most examined strains in the environment of 0.5% glucose formed weaker biofilm when compared with the medium without this carbohydrate. Analysis of the results concerning biofilm formation by strains isolated from patients and carriers indicated a decrease in its formation in the presence of glucose by 82.6% and 69.6%, respectively. A similar relationship between biofilm formation by bacteria and environmental growth condition was observed by other authors [13,14]. Stepanović et al. (2004) [13] reported that the strongest biofilm formation by *Salmonella* spp. occurred in a diluted medium with low glucose concentration, least abundant in nutrients—1/20 TSB. Perhaps, this constitutes a defensive mechanism against unfavorable environmental factors. The presence of glucose in the biofilm environment of some Enterobacterales rods reduced its formation, which is connected to catabolic repression [15,16]. This results in a decrease in expression of genes encoding type 1 fimbriae responsible for the penetration of *Salmonella* spp. into host cells or forming biofilm on infected tissues [15,16]. Jackson et al. (2002) [17] indicated that the presence of glucose in a concentration of 0.2% in the environment reduces biofilm formation on the surface of polystyrene by *Escherichia coli*, *Klebsiella pneumoniae*, *Citrobacter freundii,* and *S.* Typhimurium during 24-h incubation. Another researcher reported that the presence of glucose in the culture medium inhibits biofilm formation by the strains of *Hafnia alvei* [14], *Aeromonas hydrophila* [18], and *Citrobacter werkmanii* [19].

No effect of glucose on biofilm formation was observed by Bagge et al. (2001) [20] for *Shewanella putrefaciens*. Stimulating the effect of glucose on biofilm formation was in turn proved by other authors towards *Burkholderia pseudomallei* [21], *Staphylococcus aureus,* and *E. coli* [22]. In our study, only one strain showed an increase in the intensity of biofilm formation in an environment with glucose. The results of these experiments suggest that biofilm formation can be affected by the species of studied bacteria, properties of a strain, and the type of surface on which bacteria form biofilm.

S. *enterica* is maintained in the gallbladder, which can lead to chronic carrier status. Forming biofilm on the surface of gallstones may protect bacteria against the bactericidal effect of bile salts [23]. It was proved that bile affects the virulence of bacteria, e.g., mobility and invasion [24]. A study by Prouty et al. (2002) [25] shows that increased biofilm formation by *S.* Typhi and *S.* Typhimurium on the surface of gallstones in in vitro conditions depends on the presence of bile salts. In the present study, the inhibitory effect of 3.0% bovine bile on 24-h biofilm formation on the surface of polystyrene was indicated for 85.5% strains of *S. enterica*. In the bile environment, 60.9% of strains did not form biofilm, and the other 39.1% formed weak biofilm. Strains isolated from patients and carriers showed a decrease in biofilm formation in the presence of bile, 93.5% and 69.6%, respectively. Only for 14.5% of the studied *S. enterica* adding 3.0% of bile to the medium did not change the degree of biofilm formation. Our results are in opposition to the results obtained by Kotian et al [26]. They found that 3% bile increases biofilm formation by non-typhoidal *salmonella* serovars isolated from seafood and poultry [26]. Growth of *Salmonella* spp. in sub-lethal concentrations of bile causes changes in gene expressions, and consequently, their adaptation to bile concentrations which lead to cell death. This phenomenon may be of importance in the course of chronic infection with these pathogens [23]. The results of our study, as well as by Prouty et al. (2002) [25], allow us to suppose that biofilm formation is affected by the properties of the strain and the surface.

Biofilm formation in the gallbladder by *Salmonella* spp. reduces the effectiveness of certain antibiotics [27,28,29]. Biofilm-related infections are difficult to treat. During antibiotic therapy, most of the time microorganisms are exposed to the antibiotics at sub-MIC concentrations, because their MIC is usually maintained in the body for a short time after administration. Patients are subjected to the effects of sub-MICs due to insufficient dosages of applied antibiotics or fluctuations in drug concentrations between doses. Sub-minimal concentrations of drugs may induce changes in bacterial cell morphology. They may also affect the induction of prophages, the growth rate of bacteria, their enzymatic activity, and ability to adhere to the host cells [27,28,29].

Few works inform about the effect of antibiotics on biofilm formation by *Salmonella* spp. [8,30]. Sub-MICs of different antibiotics also inhibited biofilm formation by strains of *E. coli* [31] and *Proteus mirabilis* [32]. Other authors reported an increase in biofilm formation by Gram-positive and Gram-negative bacteria in the environment of sub-MICs of antibiotics [27,33,34].

Ampicillin penetrates well into the biliary tract. Hence, it is used in the treatment of gallbladder infections caused by *Salmonella* spp. The results of our study have shown that the values 0.125, 0.25, and 0.5 MIC of ampicillin reduce biofilm formation by these bacteria on the surface of polystyrene during a 24-h incubation. Among strains isolated from patients and carriers, a decrease in biofilm formation was found along with an increase in the value of sub-MIC of the antibiotic. The percentage of strains isolated from patients and carriers where a decrease in biofilm formation was shown amounted to 76.1% and 82.6%, respectively.

## 5. Conclusions

Presented results indicate the ability to form biofilm by *Salmonella* spp. on the surface of polystyrene. There was found a relationship between biofilm formation and the presence and concentration of glucose, bile, and the value of sub-MIC of ampicillin. The ability to form biofilm by these pathogens is varied and depends on the serovar. It can be supposed that changes in the formation of this structure shown in vitro may be also important in vivo. These characters affect adhesive properties that determine biofilm formation. One should not omit the impact of sub-MICs of antibiotics which may contribute not only to the selection of strains resistant to drugs but also to a change of virulence factors, e.g., biofilm formation.

Knowledge of the factors that determine and influence biofilm formation by *S. enterica* will allow for the development of methods that prevent or limit its formation. This, in turn, may prevent the development of infection and carrier status.

## Figures and Tables

**Table 1:** The origin of tested *S. enterica* strains (*n* = 69).

Department Type of Material	Serovar *S. enterica*								Total (*n* = 69)	
	Enteritidis (*n* = 59)		Infantis (*n* = 5)		Typhimurium (*n* = 3)		Mbandaka (*n* = 2)			
	*n*	%	*n*	%	*n*	%	*n*	%	*n*	%
The Tadeusz Browicz Provincial Hospital for Infectious Diseases and Observation in Bydgoszcz, Poland										
Feces	35	50.7	1	1.5	1	1.5	1	1.5	38	55.1
Blood	1	1.5	0	0.0	0	0.0	0	0.0	1	1.5
Antoni Jurasz University Hospital No. 1 in Bydgoszcz, Poland										
Feces	3	4.5	0	0.0	0	0.0	0	0.0	3	4.5
Blood	3	4.5	0	0.0	1	0.0	0	0.0	4	5.8
Provincial Sanitary-Epidemiological Station in Bydgoszcz, Poland										
Feces	17	24.6	4	5.8	1	1.5	1	1.5	23	33.3

**Table 2:** Biofilm of *S. enterica* strains (*n* = 69), including serovars.

Origin of Strains	Biofilm							
	Weak		Moderate		Strong		Total	
	*n*	%	*n*	%	*n*	%	*n*	%
*Salmonella* Enteritidis (*n* = 59)								
Sick People	20	47.6	18	42.9	4	9.5	42	71.2
Carriers	11	64.7	5	29.4	1	5.9	17	28.8
Total	31	52.5	23	39.0	5	8.5	59	100.0
*Salmonella* Infantis (*n* = 5)								
Sick People	1	100.0	0	0.0	0	0.0	1	20.0
Carriers	2	50.0	1	25.0	1	25.0	4	80.0
Total	3	60.0	1	20.0	1	20.0	5	100.0
*Salmonella* Typhimurium (*n* = 3)								
Sick People	2	100.0	0	0.0	0	0.0	2	66.7
Carriers	0	0.0	1	100.0	0	0.0	1	33.3
Total	2	66.7	1	33.3	0	0.0	3	100.0
*Salmonella* Mbandaka (*n* = 2)								
Sick People	0	0.0	1	100.0	0	0.0	1	50.0
Carriers	0	0.0	1	100.0	0	0.0	1	50.0
Total	0	0.0	2	100.0	0	0.0	2	100.0
Total (*n* = 69)								
Sick People	23	50.0	19	41.3	4	8.7	46	66.7
Carriers	13	56.5	8	34.8	2	8.7	23	33.3
Total	36	52.2	27	39.1	6	8.7	69	100.0

## Supplementary Materials

The following are available online at https://www.mdpi.com/2076-2607/9/1/43/s1, Figure S1: The effect of glucose on biofilm formation by *Salmonella enterica* strains (a, b, c,** values marked with different letters differ statistically significantly (*p* ≤ 0.05); Figure S2: The effect of bovine bile on biofilm formation by *Salmonella enterica* strains (a, b, c, values marked with different letters differ statistically significantly (*p* ≤ 0.05).
