# Supplementary figures and images for: Influence of Selected Factors on Biofilm Formation by Salmonella enterica Strains
Source: Microorganisms. 2020 Dec 25;9(1):43. doi: 10.3390/microorganisms9010043 (PMC7824446; doi:10.3390/microorganisms9010043)

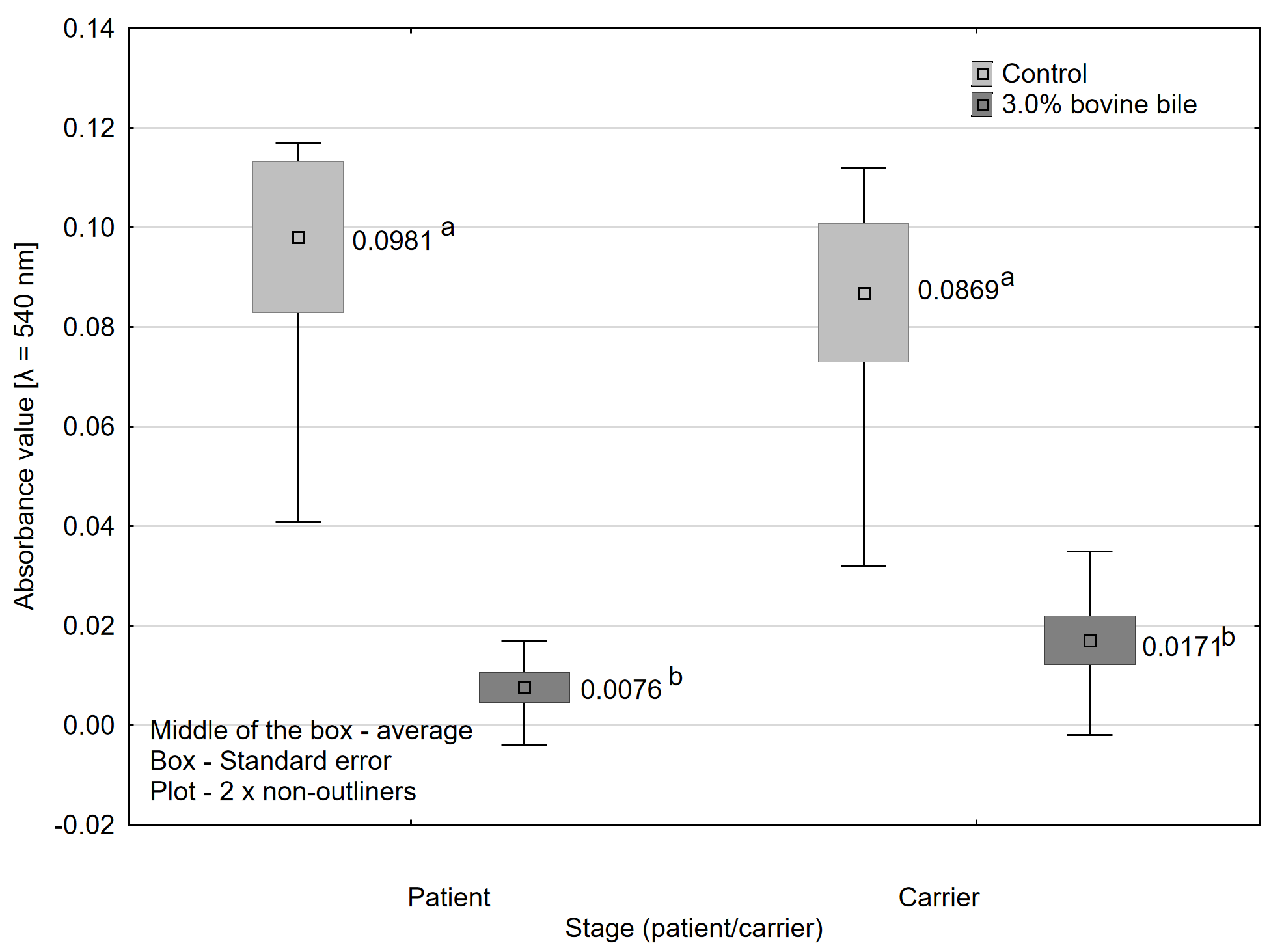

Supplement: Supplementary file 1 [file microorganisms-09-00043-s001.zip › Białucha A. et al. - Fig. 2 Supplement.tif]

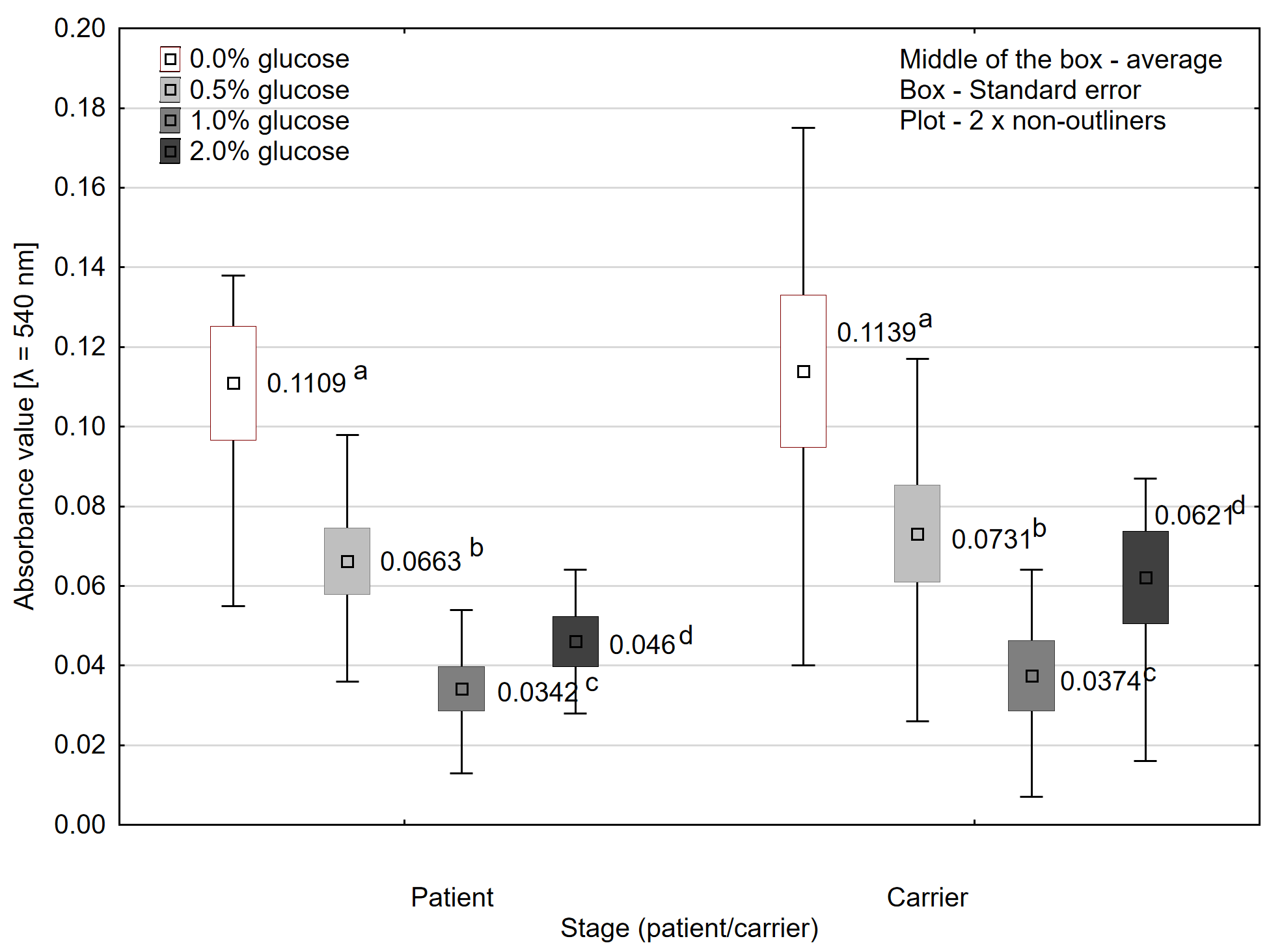

Supplement: Supplementary file 1 [file microorganisms-09-00043-s001.zip › Białucha A. et al. - Fig. 1 Supplement.tif]
